# Treatment Outcomes of Pediatric Patients With Ewing Sarcoma in a War-Torn Nation: A Single-Institute Experience From Iraq

**DOI:** 10.1200/JGO.18.00122

**Published:** 2019-02-01

**Authors:** Sazgar S. Majeed, Hawzheen A. Muhammad, Jalil S. Ali, Hassanain H. Khudhair, Ayah Said, Shkar O. Arif, Karzan M. Murad, Ali H. Gendari, Bamo M. Muhsin, Shwan A. Mohammed, Layth Mula-Hussain

**Affiliations:** ^1^ Zhianawa Cancer Center, Sulaimani, Kurdistan, Iraq; ^2^ Komar University of Science and Technology, Sulaimani, Kurdistan, Iraq; ^3^ University of Sulaimani, Sulaimani, Kurdistan, Iraq; ^4^ University of Toronto, Toronto, Ontario, Canada; ^5^ Mosul Oncology and Nuclear Medicine Hospital, Mosul, Ninevah, Iraq; ^6^ University of Alberta, Edmonton, Alberta, Canada

## Abstract

**PURPOSE:**

Ewing sarcoma (ES) is a relatively rare, highly malignant tumor of the musculoskeletal system. It is the second most common malignant bone tumor in children and adolescents in the age group of 5 to 20 years. The aim of this study was to identify the treatment outcomes of pediatric patients with ES in Sulaimani governorate, Iraq.

**PATIENTS AND METHODS:**

This was a retrospective study that reviewed the medical records of pediatric patients with ES who were managed between 2009 and 2015, with follow-up until late 2017. Patient- and tumor-related factors were correlated with clinical outcomes.

**RESULTS:**

A total of 31 pediatric patients with ES were included in this study. All the patients received chemotherapy and radiotherapy, whereas only 14 patients underwent surgical resection and just eight had free surgical margins. The median age at diagnosis was 13 years, 58% were male, and 42% were female. The presenting symptoms at diagnosis were mostly pain (67.7%) and palpable mass (25.8%). The primary tumor was located in the extremities (51.6%), the thoracic cage (19.4%), the pelvis (16.1%), and the lumbar vertebrae (12.9%). Approximately two thirds of the patients (61.3%) had localized disease at the time of presentation. The 5-year overall survival was 19%, and the 5-year recurrence-free survival was 34%.

**CONCLUSION:**

Clinical outcomes of ES in pediatric patients in our war-torn nation, Iraq, are still markedly inferior to the published outcomes from stable, developed nations. Additional large and multicenter national studies are required. Diagnostic and therapeutic measures need improvement, and multidisciplinary and comprehensive cancer-integrated approaches are vital for better outcomes.

## INTRODUCTION

Ewing sarcoma (ES) belongs to the ES family of tumors, which includes ES (osseous and extraosseous) primitive neuroectodermal tumors of the musculoskeletal tissues and malignant small cell tumors of the thoracopulmonary region (Askin tumors).^1^ There is a slight predominance of ES in the male sex (male/female ratio, 1.3:1).^2-4^ Although in general it is rare malignant disease, the ES family of tumors is the second most common primary tumor of the bone in children 5 to 20 years of age.^5^

The incidence of ES is approximately 1 in 1,000,000 children younger than 15 years of age in the United States population.^6^ In the European Intergroup Cooperative Ewing Sarcoma Study,^7^ it was shown that 24.7% of ES lesions were located in the pelvis, 16.4% in the femur, 16.7% below the knee, 12.1% in the ribs, 8.0% in the spine, and 4.8% in the humerus. It was also seen that ES of the bones usually develops in the diaphysis of the long bones.^8^

ES is an aggressive, rapidly growing malignant tumor that develops mainly in osseous sites (85%) but also in extraskeletal soft tissue.^9^ Extraskeletal ES usually originates in the soft tissues of the lower extremities, paravertebral region, chest wall, or retroperitoneum.^10^ ES spreads to the lungs, bones, and bone marrow, with poorer prognosis if metastasized to the latter two sites compared with the lung only.^11^

Histologically, ES tumors are composed of small, blue, round, uniform tumor cells that are intermixed with light cell and dark cell areas.^12^ Immunohistochemically, ES tumors express markers including cluster of differentiation 99, Friend leukemia integration 1 transcription factor, and caveolin1 that can contribute to the diagnosis of the disease.^13-15^

Currently, there is no standard staging system for ES.^16^ According to the 2013 Blueprint for Research from the Children’s Oncology Group, two stages of ES are recognized: localized and metastatic. The Children’s Oncology Group found that approximately 25% of patients had metastatic disease on clinical presentation, and this was found in the lungs (60%), bone (43%), and/or bone marrow (19%).^17^ According to the European Society for Medical Oncology Guidelines Working Group, all forms of ES are considered high-grade tumors.^18^

**CONTEXT**

Ewing sarcoma (ES) is a relatively rare, aggressive, and rapidly growing malignant tumor of the musculoskeletal system, but it is the second most common bone tumor in children and adolescents.
Clinical outcomes of pediatric patients with ES in Iraq are still inferior to other international experiences.
Diagnostic and therapeutic measures need improvement in Iraq.

The most frequent presentations of patients with ES are localized pain and a palpable mass. Pain and swelling may present for many months before diagnosis.^19^ Symptoms of systemic disease, including low-grade fever, malaise, and weakness, sometimes occur.^4^ In the clinical diagnosis of ES, a thorough history taking and physical examination are critical. The diagnostic work-up for patients with ES may comprise blood investigations, including CBC count, erythrocyte sedimentation rate, and serum lactate dehydrogenase (LDH). Studies have shown that high serum LDH in bone ES has a prognostic value.^20^ Imaging studies for ES include plain radiographs, computed tomography (CT) scanning, and magnetic resonance imaging of the primary region. A chest CT scan must be obtained to rule out lung metastases. A bone marrow biopsy or aspiration is important to exclude bone marrow involvement.^21^

Patients with ES require a multimodal treatment approach, including chemotherapy (CTX), surgery, and radiotherapy (RTX). The standard treatment involves neoadjuvant CTX (administered before any other treatment to induce tumor shrinkage) and local therapy (surgery and/or RTX), followed by adjuvant CTX. Effective local and systemic therapy is critical for better outcomes in these patients.^22^

Several factors have been shown to influence the choice of local treatment in patients with ES, such as the patient’s age and the site, size, and local extension of the tumor.^23^ Because ES is a chemosensitive disease, induction CTX is preferred to concomitant systemic and local therapy. CTX regimens have been optimized by using various cyclic combinations of drugs incorporating doxorubicin, vincristine, cyclophosphamide, ifosfamide, etoposide, and dactinomycin.^24^ Surgery with adequate safe margins, whether limb salvage or amputation, is regarded as the best treatment for local control, but it may not always be feasible. RTX may be used in combination with surgery when there is a poor response to CTX (radiologic or histologic) or concerns about safe surgical resection margins, or when the anatomic site makes complete resection impossible.^25,26^

Despite improvements in treatment, the global 5-year survival rate is approximately 70% in patients with localized disease and 30% in those with metastatic disease.^27^ Patients with ES in war-torn nations might suffer from poor treatment outcomes because of different challenges. The aim of our study was to evaluate these outcomes (survival rate and event-free survival [EFS]) in patients who were treated with CTX and local therapy (surgery and/or RTX) at the only twin facility in Iraq: Hiwa Cancer Hospital (HCH) and Zhianawa Cancer Center (ZCC); both are tertiary medical oncology, radiation oncology, in-sequence, public, free-of-charge, cancer facilities in Sulaimani governorate, the fourth largest population-based governorate in Iraq (after Baghdad, Ninevah, and Basra).

## PATIENTS AND METHODS

After obtaining approval from the ethics and research committee at the University of Sulaimani, on May 9, 2017, this project was launched. It is a retrospective study via medical record review of medical data from 31 patients diagnosed with ES and managed in HCH–ZCC with local surgical facilities throughout the country during the period of March 2009 to December 2015, with outcomes followed up until August 2017. Patients with ES were categorized on the basis of sex, age at diagnosis, residency (inside or outside Sulaimani city), primary tumor site, tumor size, year of diagnosis, stage at diagnosis, clinical presentation, information regarding having received CTX or not, surgery performed or not, intention of RTX (curative or palliative), and relapse or recurrence of the disease. For the purpose of organizing the data, a questionnaire was prepared (Appendix). The questionnaire was filled out by the research team on the basis of direct contact with the patients and/or their guardians.

During the process of data collection, patients or their families were contacted for follow-up until August 2017. Accordingly, the general condition of the patient was evaluated and recorded as either being alive or dead (with the date of death). The total number of patients diagnosed with ES in the HCH database in that period of time was 40 patients, but only 31 of them were referred to ZCC, and their data are complete. The remaining nine patients could not be included in this study because they did not receive treatment at HCH and traveled outside the country after registering at HCH.

Because of the unavailability of cytogenetic testing in our region, the diagnosis of ES was based on histopathologic microscopic findings and immunostaining only. Most of the patients underwent CT at the primary tumor site, and all had bone marrow aspirates and biopsies. Some of the patients also had positron emission tomography scans. For follow-up, the patients were evaluated every 4 to 6 months via clinical assessment and imaging.

Patients who have indications for RTX are usually prepared for CT simulation according to the site of the cancer, in the supine or prone position, using a vacuum bag to decrease movement. After the CT simulation, an RTX plan is organized by a medical physicist and is reviewed and approved by the radiation oncologist; the treatment is delivered by radiation therapists. A total of 55.8 Gy/31 fx for definitive purposes and 50.4 Gy/28 fx for postoperative purposes is the range of the RTX dose. A CT simulator (Optima 580; GE Healthcare, Chicago, IL) and a linear accelerator, LINAC (Elekta Synergy, Stockholm, Sweden) are used. Children younger than 5 years of age are treated under general anesthesia.

### Statistical Analysis

Patient data were analyzed by SPSS software version 21 (SPSS, Chicago, IL). χ^2^ tests were used to compare the categorical data between living and dead patients, and the Kaplan-Meier method was used for survival curves. We defined overall survival (OS) as the time from the date of diagnosis until death or last follow-up, recurrence-free survival (RFS) as the time from the date of starting treatment to the date of disease recurrence, and EFS as the time from the date of starting treatment to the date of the event. *P* values of ≤ .05 were used as a cutoff point for significance.

### Inclusion Criteria

Iraqi patients 18 years or younger who had had a histopathologic diagnosis of ES between March 2009 and December 2015 and were treated at HCH and ZCC were included in the study.

### Exclusion Criteria

Patients older than 18 years of age and those who registered at HCH but initiated disease management outside the country were excluded in the study.

## RESULTS

### Patients, Tumors, and Treatments

Thirty-one pediatric patients diagnosed with ES (21 osseous and 10 extraosseous) were included in this study. Sociodemographic data, including patient, tumor, and treatment characteristics are listed in Table 1. No available information about LDH nor the treatment protocols were available for these patients..

**TABLE 1:**
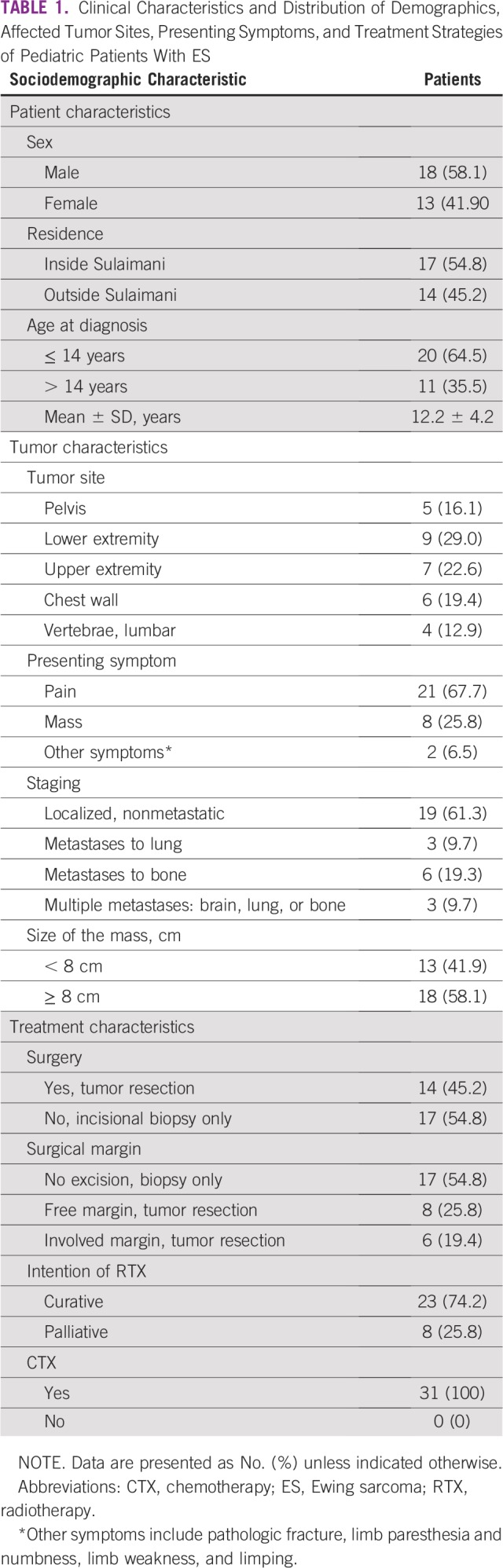
Clinical Characteristics and Distribution of Demographics, Affected Tumor Sites, Presenting Symptoms, and Treatment Strategies of Pediatric Patients With ES

### Tumor Recurrence or Death

A total of 61.3% of patients had a recurrence, with 48.4%, 9.7%, and 3.2% having a recurrence as metastatic disease to the bones, multiple organs (brain, bones, and lungs), and lungs only, respectively. Results from this study have shown that 20 patients (64.5%) died at the end of the study and 11 patients (35.5%) were alive at the time of last follow-up (Table 2).

**TABLE 2:**
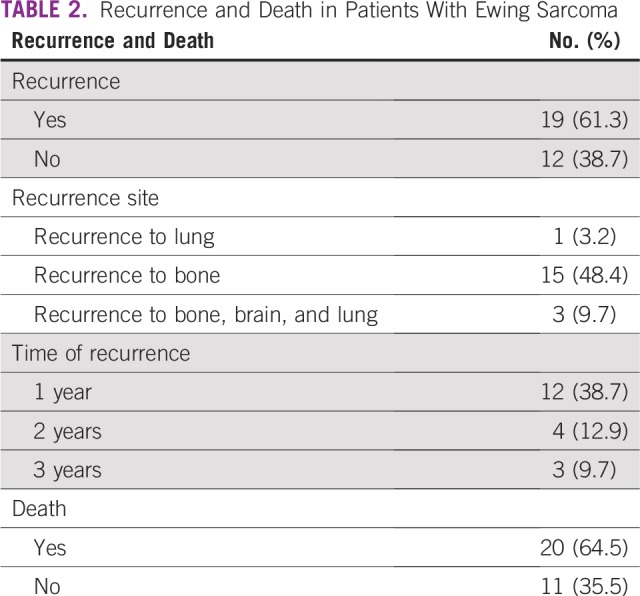
Recurrence and Death in Patients With Ewing Sarcoma

### Survival Rates in Patients With ES

**FIG 1:**
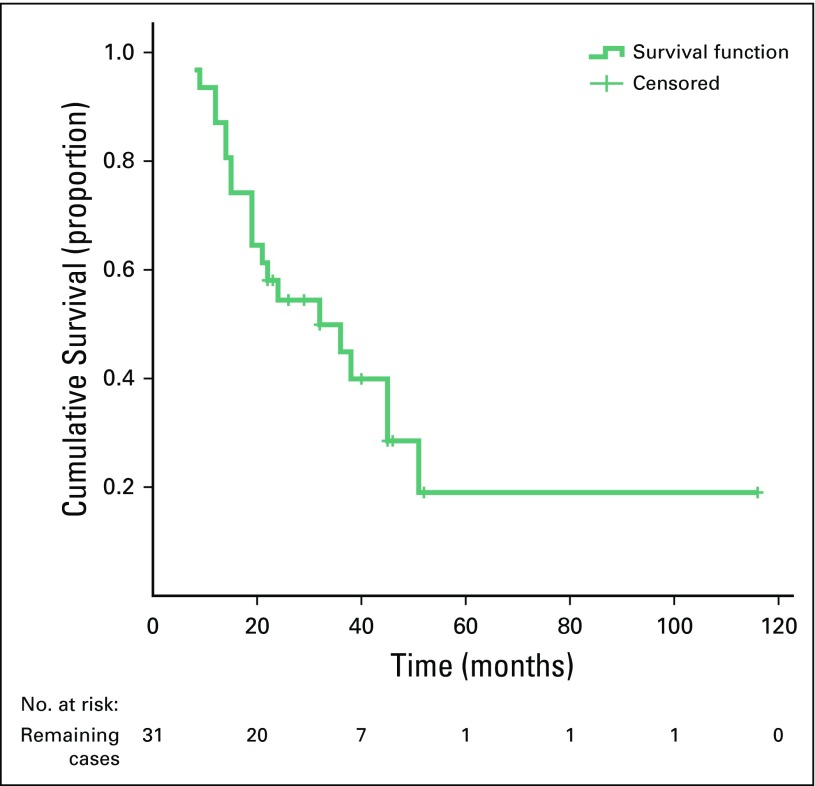
Kaplan-Meier curve showing overall survival.

**FIG 2:**
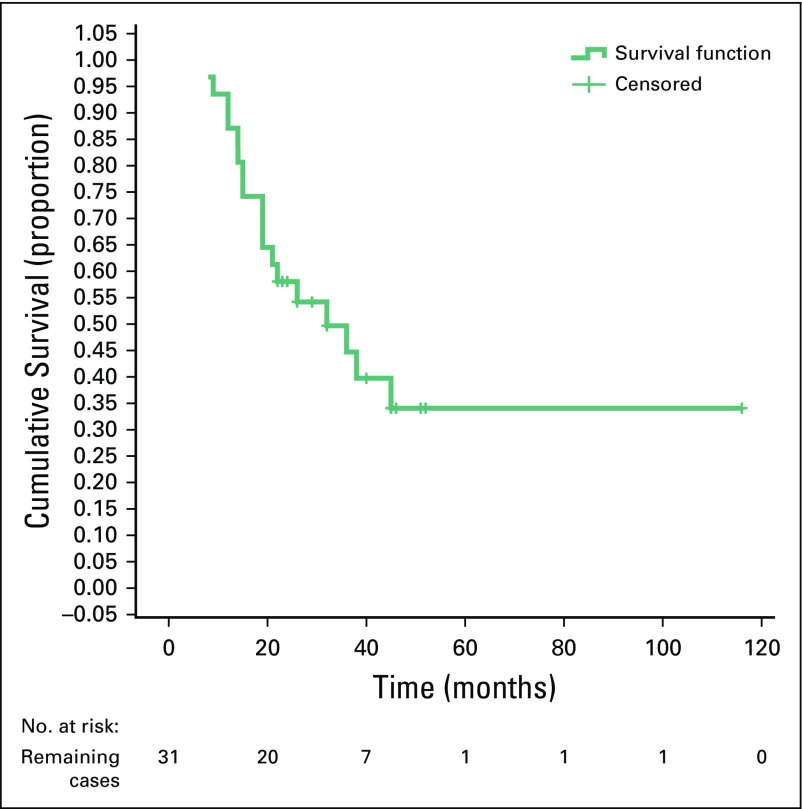
Kaplan-Meier curve showing recurrence-free survival.

**FIG 3:**
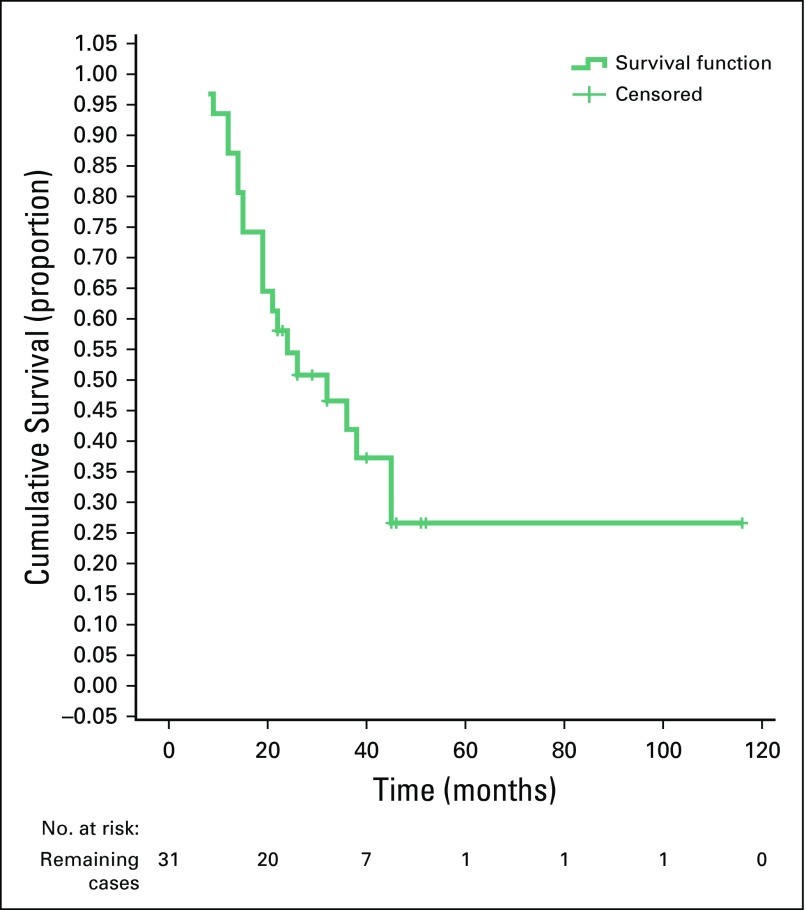
Kaplan-Meier curve showing event-free survival.

There was a statistically significant difference between the survival rate and the sex of the patients (poor survival was seen in female patients; *P* = .047), and a highly significant difference between poor survival rates and recurrence of the disease (*p* ≤ .001). However, there were no statistically significant differences between survival rates and other variables (Table 3). Furthermore, there was a significant difference between disease recurrence and mean survival (MS) time. For recurrence, MS was 22.3 months (95% CI, 16.4 to 28.1 months) and for no recurrence, MS was 90.8 months (95% CI, 60.4 to 121.3 months; Table 4). There were no differences in the MS time and other variables. The 3- and 5-year OS were approximately 45% and 19%, respectively (Fig 1), whereas the 5-year RFS and EFS were 34% and 25%, respectively (Figs 2 and 3).

**TABLE 3:**
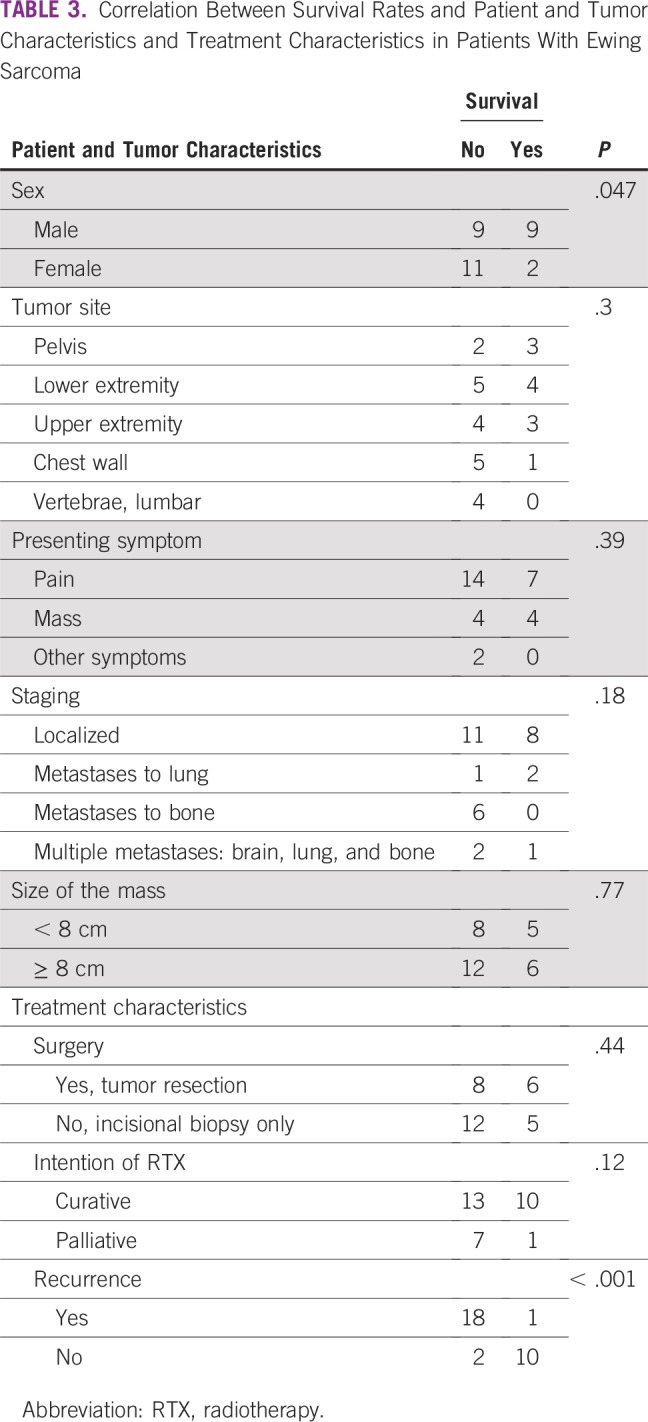
Correlation Between Survival Rates and Patient and Tumor Characteristics and Treatment Characteristics in Patients With Ewing Sarcoma

**TABLE 4:**
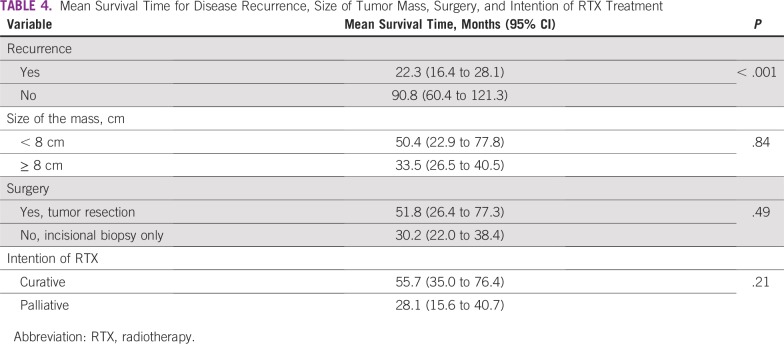
Mean Survival Time for Disease Recurrence, Size of Tumor Mass, Surgery, and Intention of RTX Treatment

## DISCUSSION

To our knowledge, this is the first study of the treatment outcomes of ES in Iraq, a war-torn nation for the last 3 to 4 decades. Our patients’ ages ranged from 1 to 18 years (more than one half were 14 years or younger), and the median age was 13 years, which is close to that of other studies. According to the literature, age is a prognostic factor, with poorer outcome associated with older age at presentation (≥ 14 years).^28^ Regarding sex predominance, we observed a slight male predominance, in agreement with the findings of other studies.^29,30^

We found that the most common anatomic sites were the lower extremities, followed by the chest wall and other sites (lower extremity in 45%, pelvis in 20%, upper extremity in 13%, axial skeleton and ribs in 13%, and face in 2%), which is in agreement with some reported studies.^16^ However, there is some controversy regarding these findings.^5,8,18,19^ As for the presenting symptoms, pain was reported to be the most common complaint, followed by swelling, limb paresthesia, weakness, and limping, which is in parallel with the findings of other studies.^31,32^

More than one half of our patients with ES had localized disease, but at the time of diagnosis, 12 patients (38.7%) presented with metastatic disease, with most being metastatic to bone followed by the lungs or multiple sites. According to the literature, metastases at diagnosis is one of the poorest prognostic factors, and this may be part of the explanation for the poor survival in our patients, of whom more than one third were diagnosed with metastatic disease. Cotterill et al^7^ reported that only 22% of patients with ES had metastatic disease at diagnosis. Metastasis at diagnosis can be multifactorial; it can be related to the time interval between diagnosis and the start of treatment. A delay in diagnosis can be another factor.

An ES tumor with a diameter of ≥ 8 cm is regarded as a poor prognostic factor. Therefore, on this basis, we grouped the sizes of the tumors into < 8 cm (small) or ≥ 8 cm (large). Although the correlation between tumors ≥ 8 cm and OS was not statistically significant, it was found in more than one half of our patients. The significance of tumor size has been documented in previous studies as a significant predictor factor of worse OS.^3,33^ These studies support our findings and this may further explain our poor OS.

Regarding treatment, all of the 31 patients included in this study were treated with CTX and RTX, and the intention of RTX was either curative (in 74.2% of patients, whether as definitive or adjuvant) or palliative (in 25.8% of patients). Seventeen patients underwent incisional biopsies only, and 14 also had surgical excision. Among the latter, only eight ended up with free surgical margins and six, unfortunately, had involved margins. Because of a lack of detail regarding the CTX dosages and protocols in the archives, we were unable to report these details and their impact on the treatment outcome. In addition, we could not discern a statistical significance between RTX and the survival rates, which is, in fact, in disagreement with a study that showed that RTX use improved local control and was associated with improved PFS.^34^

Most of our patients (61.3%) had recurrent disease as metastases. Recurrence was found to be statistically significant factor (*p* < 0.001) for decreasing survival. In agreement with this, Stahl et al^35^ analyzed the risk of recurrence and survival after relapse, the type and time of relapse, and OS after relapse in 714 patients. The patients were treated within the Cooperative Ewing Sarcoma Study 81 or 86 or the European Intergroup Cooperative Ewing Sarcoma Study 92. Recurrences were shown to be associated with poor survival in patients with ES. Among our patients with recurrent disease, 38.7% had a recurrence within the first year, 12.9% had a recurrence within the second year, and 9.7% had a recurrence within the third year from the time of diagnosis. Similar to this, the findings of a study by Leavey et al^36^ showed that those with earlier recurrences ended up with poorer survival.

Unfortunately, our 3- and 5-year OS were 45% and 19%, respectively, whereas the 5-year RFS and EFS were 34% and 25%, respectively. These outcomes were relatively comparable to a study with 32 patients with ES in a neighborhood country, Iran, in which the 5-year DFS was 26%, mean OS was 38.7 months, and 5-year OS was 25% and the presence of metastases at diagnosis had a significant effect on OS.^37^

ES is an aggressive and rapidly growing malignant tumor that can become metastatic early in the disease, meaning treatment may not be effective. From this study in a war-torn nation, Iraq, we conclude that the survival rates of patients with ES are poorer than in stable, developed nations. However, this is a single-institute study with a small number of patients. Larger national studies from multiple centers with longer follow-up times are required to determine more exactly the key areas crucial to improving treatment outcome in patients with ES. Current methods of diagnostic and therapeutic measures seem to be ineffective in improving outcomes. Access to better equipped and staffed multidisciplinary clinics and improving our diagnostic and therapeutic measures to be in parallel with international standards are critical and are warranted to improve our outcomes.

## Appendix

### Questionnaire

Sample of questionnaire form used to collect data and assess pediatric patients with Ewing sarcoma included in this study:

Sex: male or female (1 or 2)

Age at diagnosis

Residency: inside Sulaimani or outside Sulaimani (1 or 2)

Tumor site: pelvis, lower extremity, upper extremity, chest wall, or vertebrae (1, 2, 3, 4, or 5)

Presenting symptom: pain, mass or swelling, or other symptoms (1, 2, or 3)

Tumor staging: localized or metastatic (1 or 2)

Size of the tumor mass (cm): < 8 cm or ≥ 8 cm (1 or 2)

Intention of radiotherapy: curative or palliative (1 or 2)

Surgery: yes, tumor resection, or no, incisional biopsy only (1 or 2)

Chemotherapy: yes or no (1 or 2)

Recurrence or not (1 or 2)

Time of recurrence (months)

Status of the patient at the time of last follow-up: dead or alive (1 or 2)
